# Close Association Between Platelet Biogenesis and Alveolarization of the Developing Lung

**DOI:** 10.3389/fped.2021.625031

**Published:** 2021-05-07

**Authors:** Xueyu Chen, Junyan Zhong, Dongshan Han, Fang Yao, Jie Zhao, Gerry. T. M. Wagenaar, Chuanzhong Yang, Frans J. Walther

**Affiliations:** ^1^Laboratory of Neonatology, Department of Neonatology, Affiliated Shenzhen Maternity and Child Healthcare Hospital, Southern Medical University, Shenzhen, China; ^2^Department of Neonatology, Shenzhen Maternity and Child Healthcare Hospital, The First School of Clinical Medicine, Southern Medical University, Shenzhen, China; ^3^Faculty of Science, VU University Amsterdam, Amsterdam, Netherlands; ^4^Department of Pediatrics, David Geffen School of Medicine, University of California, Los Angeles, Los Angeles, CA, United States; ^5^The Lundquist Institute for Biomedical Innovation at Harbor-UCLA Medical Center, Torrance, CA, United States

**Keywords:** lung development, neonatal lung injury, mean platelet volume, platelet distribution width, hyperoxia, platelet counts

## Abstract

Bronchopulmonary dysplasia (BPD) is a neonatal chronic lung disease characterized by an arrest in alveolar and vascular development. BPD is secondary to lung immaturity, ventilator-induced lung injury, and exposure to hyperoxia in extremely premature infants, leading to a lifelong impairment of lung function. Recent studies indicate that the lung plays an important role in platelet biogenesis. However, the dynamic change of platelet production during lung development and BPD pathogenesis remains to be elucidated. We investigated the dynamic change of platelet parameters in extremely premature infants during BPD development, and in newborn rats during their normal development from birth to adulthood. We further studied the effect of hyperoxia exposure on platelet production and concomitant pulmonary maldevelopment in an experimental BPD rat model induced by prolonged exposure to hyperoxia. We detected a physiological increase in platelet count from birth to 36 weeks postmenstrual age in extremely premature infants, but platelet counts in extremely premature infants who developed BPD were persistently lower than gestational age-matched controls. In line with clinical findings, exposure to hyperoxia significantly decreased the platelet count in neonatal rats. Lung morphometry analysis demonstrated that platelet counts stabilized with the completion of lung alveolarization in rats. Our findings indicate a close association between platelet biogenesis and alveolarization in the developing lung. This phenomenon might explain the reduced platelet count in extremely premature infants with BPD.

## Introduction

Bronchopulmonary dysplasia (BPD) is one of the most common complications of prematurity and can lead to chronic lung disease with long-term respiratory insufficiency (1). Despite advances in perinatal care, BPD continues to affect up to 40% of extremely premature infants (2). BPD is histologically characterized by arrested lung development as a result of a complex process in which lung immaturity, ventilator-induced lung injury, and exposure to hyperoxia play major roles (3). There is a growing body of knowledge about the various mechanisms underlying BPD pathogenesis (4). However, therapeutic approaches based on these mechanisms are far from being effective in clinical practice, indicating that these mechanisms probably do not operate in isolation. As the pathogenesis of lung damage in infants with BPD is not completely understood, other possible causal factors need to be elucidated (5).

Recent studies elegantly unravel the close interplay between platelet biogenesis and lung development. Tsukiji et al. report that platelet-derived CLEC-2 signals activate platelets through spleen tyrosine kinase, inducing the release of TGF-β driving the differentiation of mesothelial cells into alveolar duct myofibroblasts that are critical to primary septum formation and elastogenesis in alveolarization of the lung (6). Rafii et al. demonstrated that activated platelets release stromal-cell-derived factor and stimulate the expression of SDF-1 receptors on pulmonary capillary endothelial cells, subsequently enhancing the proliferation of alveolar epithelial cells and neo-alveolarization (7). The lungs are a major site for platelet biogenesis in humans and rodents and contribute ~50% of the total platelet production (8–12).

Little is known about the dynamic changes in platelets during the complex process that leads to BPD in extremely premature infants. Besides, the impact of clinical oxygen supplementation exposure on platelet biogenesis is unclear. We hypothesize that platelets play an important role in normal lung development and that disturbed platelet biogenesis might contribute to disrupted lung development and BPD pathogenesis. In this study, we first investigated the dynamic change of platelet parameters in a cohort of extremely premature infants within the time window of BPD development, and in newborn rats during their normal development until young adults. We also used an experimental BPD rat model to evaluate the effect of hyperoxia exposure on platelet production and aberrant pulmonary development.

## Methods and Materials

### Clinical Study

A retrospective study was performed at the Neonatal Intensive Care Unit (NICU) of the Shenzhen Maternity and Child Healthcare Hospital after approval by the Institutional Ethical Committee [SFYLS (2019)-119]. The acquirement of informed consent was waived given that no personal data were explicitly reported. Since premature infants with a younger gestational age have an increased chance to develop BPD, we only included extremely premature infants with a gestational age ≤28 weeks and/or a birth weight ≤1,000 grams. BPD was diagnosed as a requirement of supplemental oxygen at 36 weeks' postmenstrual age or discharge.

Platelet parameters were collected from complete blood counts (CBC) in the 1st week, 2nd week, 4th week, and 8th week after birth. CBC testing was performed on a Mindray 5390 analyzer (Shenzhen, China), using blood samples obtained from arterial and venipuncture or a central catheter.

### Animal Study

All animal procedures in this study were approved by the Institutional Animal Care and Use Committee of Shenzhen Institutes of Advanced Technology of the Chinese Academy of Sciences. Newborn pups from 9 pregnant Wistar rats were randomized into 8 groups: an experimental BPD group (*N* = 10) and 7 control groups (raised in room air, *N* = 10 for each group) sacrificed on postnatal day 3, 6, 10, 20, 30, 60, and at adulthood (day 90). Experimental BPD was induced by hyperoxia exposure as previously reported (13). Briefly, newborn pups were raised in a Plexiglas chamber filled with 95% oxygen for 10 days. Pups were anesthetized at the designated day by intraperitoneal injection of pentobarbital (40 mg/kg). All blood samples were drawn from the abdominal aorta, mixed with EDTA and analyzed using a Mindray 5390 analyzer (Shenzhen, China) to acquire platelet parameters. Lung tissue was fixed *in situ* under constant pressure of 27 cmH_2_O for 6 min with formalin as previously reported (13). Hereafter, the thorax was opened, the lungs were removed, fixed additionally in formalin for 24 h, embedded in paraffin and sectioned for hematoxylin and eosin (HE) staining.

#### Lung Morphometry

Mean linear intercept (MLI) was used to assess lung development status. At least 1,000 alveoli per animal were measured to calculate the MLI. Briefly, 10 non-overlapping photos of lung tissues were made with an Olympus CX43 microscope (Tokyo, Japan) at 200x magnification. Structures, including big vessels and airways, were excluded. The photos were applied for alveolar diameter analysis using a WZCamera S50 software (Shenzhen, China). Alveoli with an area of more than 100 μm^2^ were analyzed and simulated to circles for calculation of the absolute alveolar diameter. Two independent researchers blinded to the hyperoxia exposure performed the analysis.

### Statistics

Continuous parameters were displayed as mean ± standard deviation or median [interquartile range (IQR)], and analyzed by student *t*-test or Mann-Whitney *U*-test, as appropriate. Categorical variables were displayed with numbers and percentages, and analyzed by Chi-square or Fisher's exact test correspondingly. The patients' data were analyzed using SPSS statistical software version 24.0 (IBM Corporation, NY), and the animals' data were analyzed using GraphPad Prism version 8 software package (San Diego, CA, USA). A *p* < 0.05 was considered statistically significant.

## Results

### Clinical Characteristics of the Patients

**Figure 1:**
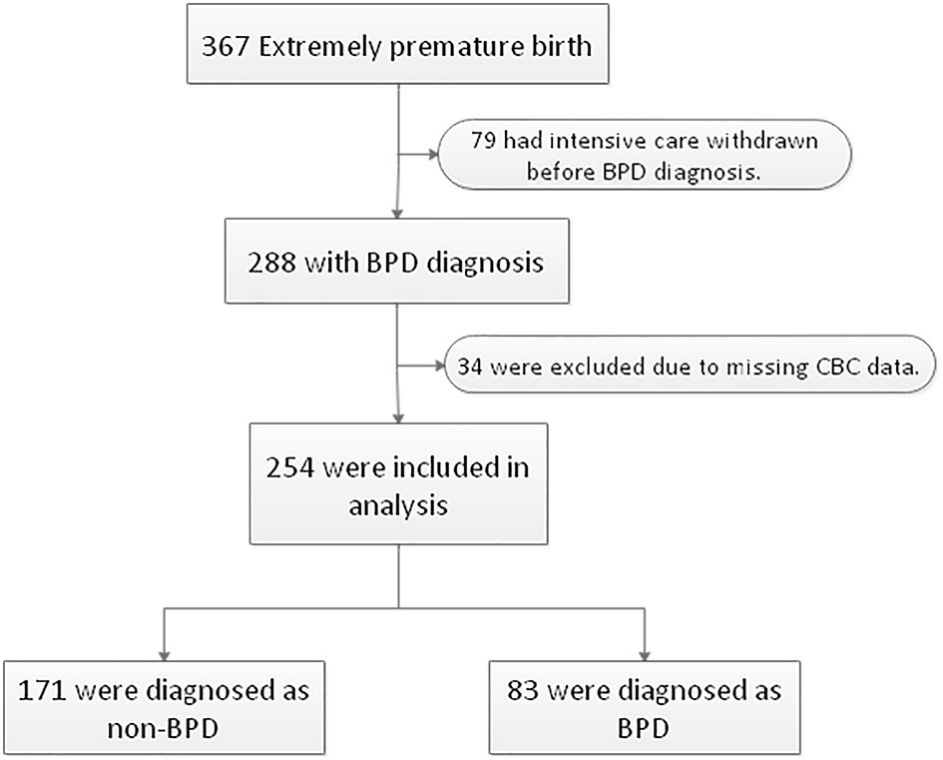
Flowchart of case selection and analysis. Two hundred fifty-four extremely premature infants were enrolled in this study. BPD, bronchopulmonary dysplasia. CBC, complete blood counts.

A total of 367 inborn extremely premature infants were admitted to our NICU during the study period. Seventy-nine infants were excluded due to life-support withdrawal before the diagnosis of BPD. Thirty-four infants were excluded due to incomplete CBC data. The remaining 254 extremely premature infants were included in the analysis, of whom 225 (88.6%) were born before 28 weeks and 29 (11.4%) were born after 28 weeks with a birth weight lower than 1,000 grams. The diagnosis of BPD was made in 83 (32.7%) infants (Figure 1). The median gestational age was 27.0 (interquartile range: 26.1–27.5) weeks, the median body weight was 910.0 (interquartile range: 807.5, 1013.2) grams. The clinical characteristics of 254 infants by BPD diagnosis were summarized in Table 1.

**Table 1:** Clinical characteristics of 254 extremely premature infants by BPD status.

	**Control (*N =* 171)**	**BPD (*N =* 83)**	***Z*/*t*/χ^**2**^**	***P-*value**
Gestational Age [Wk, M(Q1,Q3)]	27.1 (26.2, 27.6)	26.3 (25.5, 27.2)	−4.01	<0.001
Birth Weight [gr, M(Q1,Q3)]	930 (842, 1,060)	858 (720, 950)	−4.378	<0.001
Sex (male)	91	49	0.765	0.382
Gestational Diabetes Mellitus (GDM)	21	7	0.843	0.359
Gestational Hypertension (GH)	18	7	0.276	0.600
Antenatal steroid	134	66	0.094	0.760
PPROM	56	35	2.375	0.123
Twin	48	28	0.855	0.355
Delivery (C-section)	60	28	0.045	0.832
AS^1min^ [score, M(Q1,Q3)]	8 (5, 9)	6 (5, 8)	−2.049	0.040
AS^5min^ [score, M(Q1,Q3)]	10 (9, 10)	10 (9, 10)	−1.015	0.310
Surfactant	124	76	11.250	0.001
Early onset sepsis (EOS)	75	55	11.959	0.003
Intubation	83	64	21.826	<0.001
Duration of intubation (Days)	0 (0,1)	3.6 (0.8, 22)	−4.417	<0.001
Duration of CPAP (Days)	16 (7, 30)	23 (14, 41)	−3.664	<0.001
Duration of supplemental oxygen (Days)	24 (13, 41)	50 (32, 71)	−6.470	<0.001

*CPAP, continuous positive airway pressure. Continuous parameters were displayed as median [interquartile range (IQR)], and analyzed by Mann-Whitney U-test. Categorical variables were displayed with numbers and analyzed by Chi-square test. The statistics were performed using SPSS statistical software version 24.0 (IBM Corporation, NY)*.

### Dynamic Change of Platelet Parameters During BPD Development

**Figure 2:**
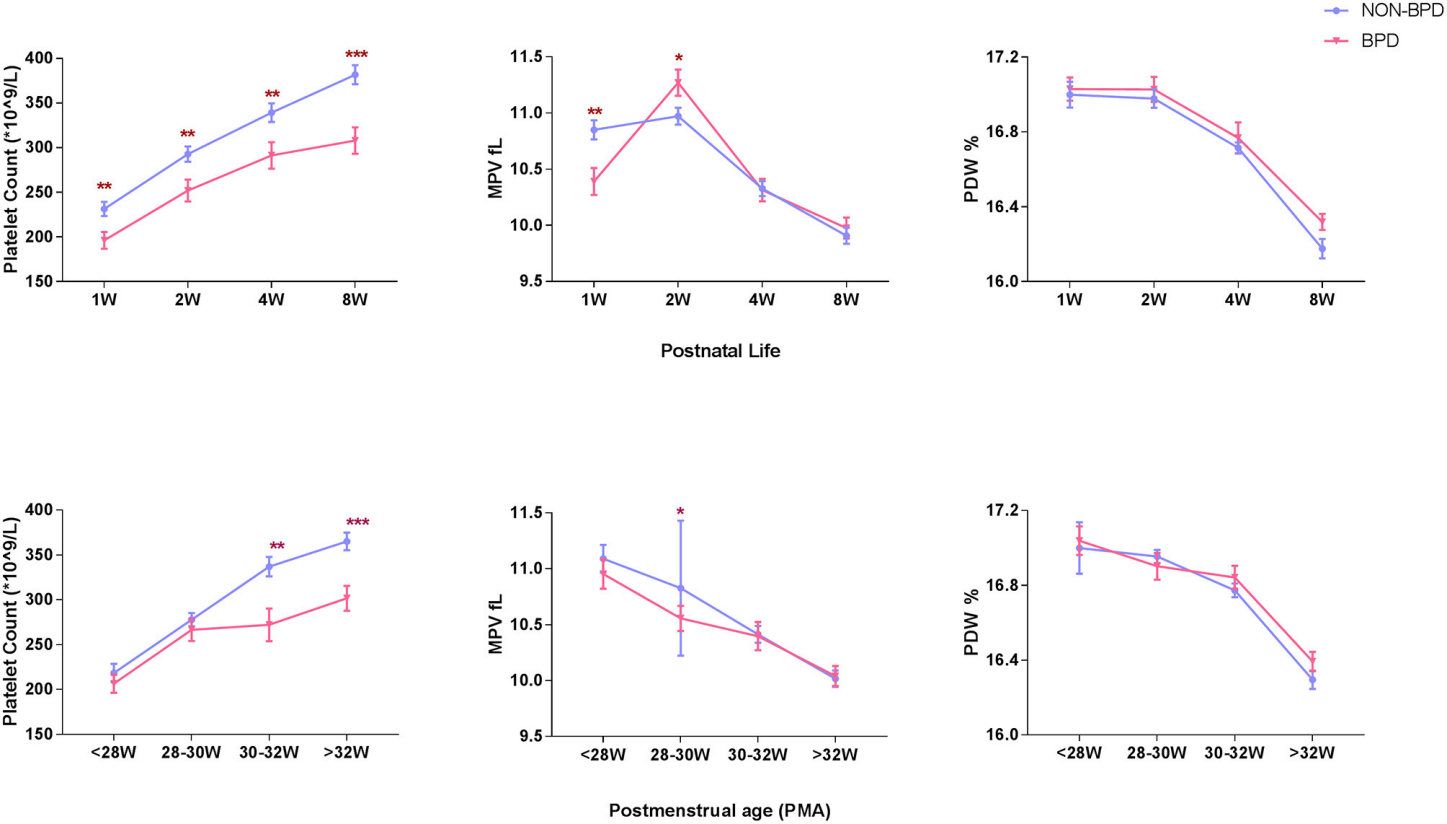
Longitudinal change of platelet parameters defined by postnatal age and postmenstrual age in BPD infants (*N* = 83) and non-BPD infants (*N* = 171). PLT, platelet count, 10^9^/L, **(A)**. MPV, mean platelet volume, fL, **(B)**. PDW, platelet distribution width, %, **(C)**. Values are expressed as mean ± SD. *p < 0.05, **p < 0.01, ***p < 0.001, compared with age-matched non-BPDs using the student *t*-test.

A total of 254 extremely premature infants were included in the analysis. BPD was diagnosed in 83 (32.7%) infants. Platelet counts (PLT) continuously increased during the first 8 weeks of postnatal life, while mean platelet volume (MPV) and platelet distribution width (PDW) showed a tendency to decline. However, platelet counts at consecutive time-points of analysis were significantly lower in infants developing BPD compared to infants without BPD (196 ± 85 vs. 231 ± 103 × 10^9^/L in the 1st week, 252 ± 112 vs. 293 ± 112 × 10^9^/L in the 2nd week, 291 ± 136 vs. 339 ± 136 × 10^9^/L in the 4th week, and 308 ± 134 vs. 382 ± 135 × 10^9^/L in the 8th week). MPV showed striking changes over the first 2 weeks, with lower values in the 1st week and higher values in the 2nd week in BPD infants compared to infants without BPD (10.39 ± 1.10 vs. 10.85 ± 1.11 fl in the 1st week, 11.27 ± 1.07 vs. 10.97 ± 0.98 fl in the 2nd week. No significant differences in PDW were observed in the two groups (Figure 2). In addition, we observed increased PLT and decreased MPV and PDW with advancing postmenstrual age (PMA). BPD infants had lower PLT after PMA of 30–32 weeks than non-BPDs (Figure 2).

### Rats With Hyperoxia-Induced BPD had Lower Platelet Counts

**Figure 3:**
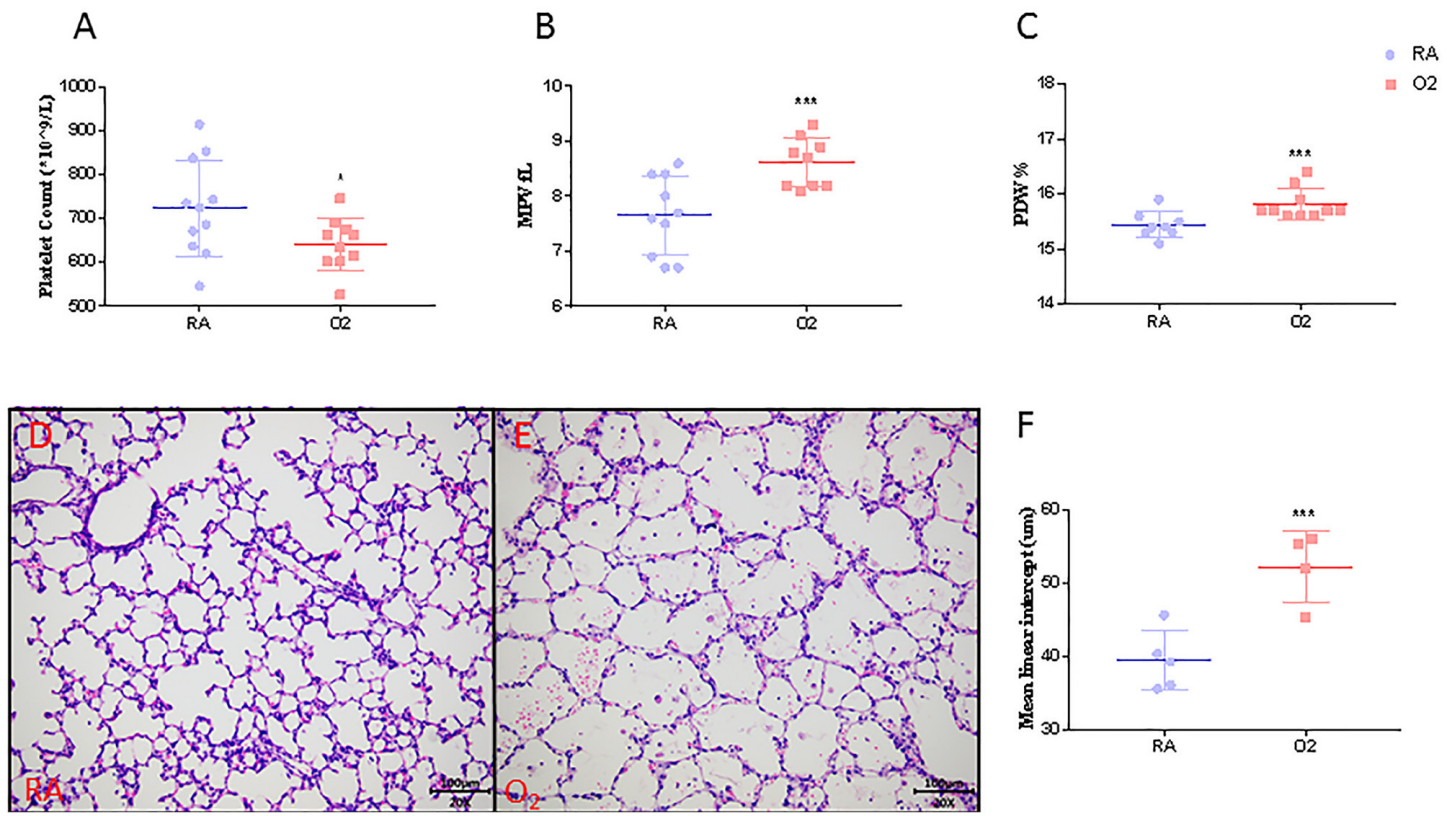
Platelet parameters (*N* = 9–10, **A–C**), mean linear intercept (MLI, *N* = 4–5, **F**) and representative photos of rat pups exposed to room air (RA, **D**) or hyperoxia (O_2_, **E**) at postnatal Day 10. Values are expressed as mean ± SD. *p < 0.05, ***p < 0.001, compared with age-matched RA controls using the student *t*-test. Magnification, 200 x.

MLI, an indicator of alveolar size, was significantly higher in rat pups exposed to hyperoxia compared to their age-matched room air (RA) controls (52.1 ± 2.4 vs. 39.5 ± 1.3, *p* < 0.001, Figures 3D–F). Similar to our clinical findings shown above, PLT were significantly lower in hyperoxia-induced BPD rats compared to controls raised in room air (642 ± 19 vs. 725 ± 23, *p* = 0.0344). MPV and PDW were significantly higher in experimental BPD pups compared to the controls (8.93 ± 0.35 vs. 7.68 ± 0.17, *p* = 0.001 and 15.81 ± 0.09 vs. 15.43 ± 0.04, *p* < 0.001, respectively, Figures 3A–C).

### Physiological Increase of Platelet Count Synchronized With Alveolarization in Rat Lung

**Figure 4:**
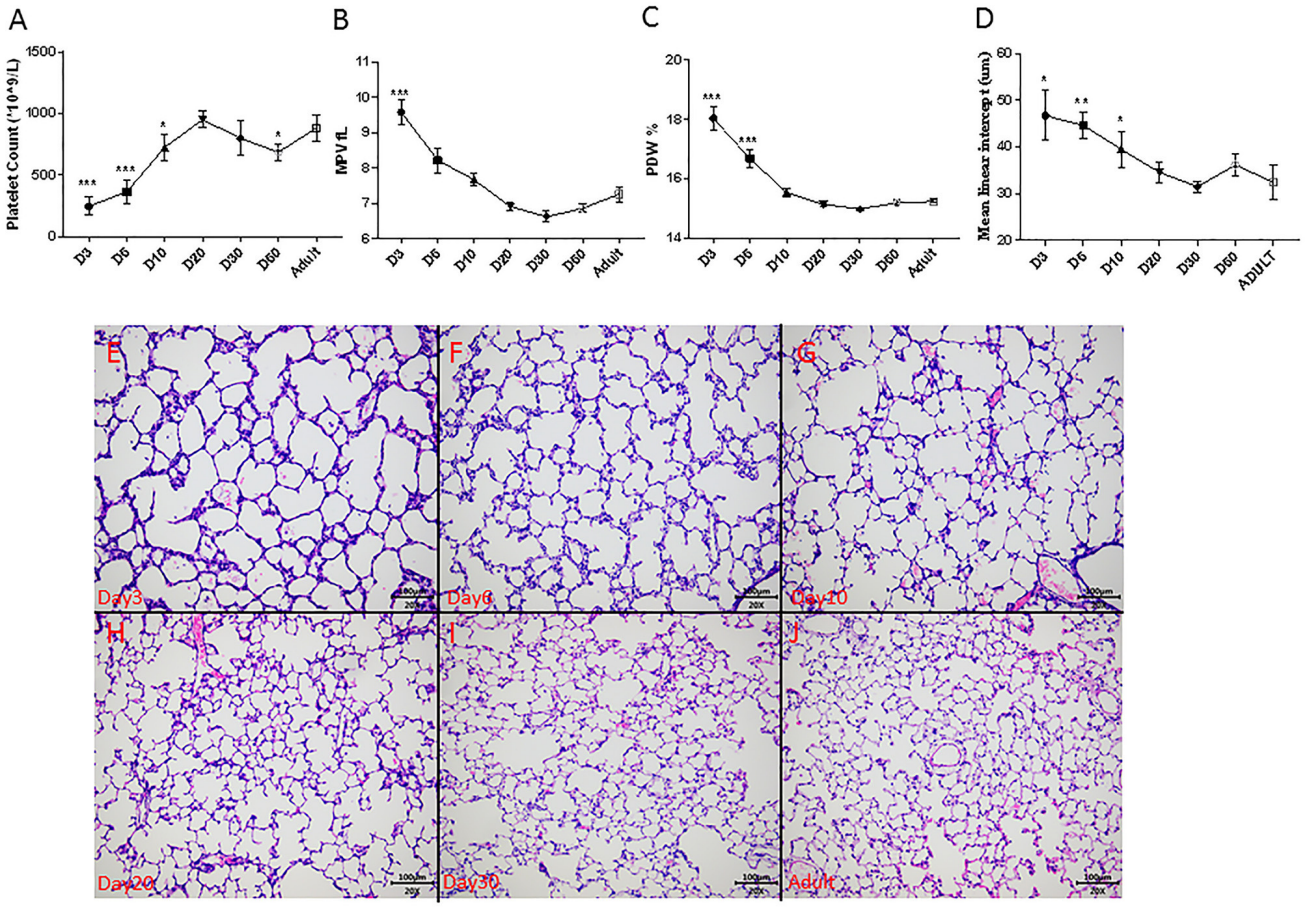
Dynamic change of platelet parameters during lung alveolarization (*N* = 8–10, **A–C**), mean linear intercept (MLI, *N* = 3–5, **D**) and representative photos during rats' development **(E–J)** in normoxia. Values are expressed as mean ± SD. *p < 0.05, **p < 0.01, ***p < 0.001, compared with adults using one-way ANOVA. Magnification, 200x.

In normal rat development, platelet counts increased from 246 ± 74 × 10^9^/L on day 3 to near-adult level of 950 ± 66 × 10^9^/L on postnatal day 20 (Figure 4A). The MPV and PDW persistently decreased to near-adult levels on postnatal day 20 (Figures 4B,C). Alveolarization of rat lung also advanced with age and was complete at around postnatal day 20, as indicated by the stabilization of the MLI (Figure 4D) and histology (Figures 4E–J).

**Figure 5:**
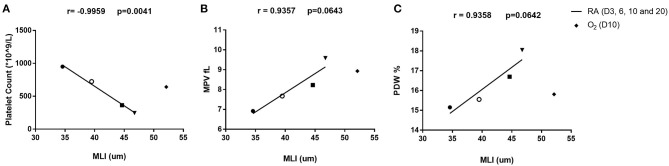
Correlation analysis between platelet parameters and MLI in rats on day 3, 6, 10, and 20 after birth. Correlation between platelet count (PLT) and MLI **(A)**, mean platelet volume (MPV) and MLI **(B)**, platelet distribution width (PDW) and MLI **(C)**. The correlation was analyzed by Pearson correlation using GraphPad Prism version 8. Triangles (▾) indicate 3-day old rats. Squares (■) indicate 6-day old rats. Open circles (°) indicate 10-day old rats and solid circles (•) indicate 20-day old rats. Diamonds (♦) stand for rats exposed to hyperoxia for 10 days in a row. The hyperoxia data were only plotted in the figure but not included in the correlation analysis.

To better depict the relationship between MLI and platelet parameters, we performed a correlation analysis on MLI and platelet indexes from 3, 6, 9, and 20 days old rats. A significant negative correlation (*r* = −0.9959, *p* = 0.0041) was observed between MLI and PLT. MPV and PDW showed a tendency toward positive correlations with MLI (*p* = 0.0643 and *p* = 0.0642, respectively, Figure 5).

## Discussion

In this study the dynamic change of longitudinal platelet parameters in premature infants and neonatal rats was investigated. We also evaluated the associations between platelet parameters and lung alveolarization in neonatal rats and found that platelet counts were significantly lower in clinical and experimental BPD. After birth, platelet counts showed a physiological increase in neonatal infants and rat pups while MPV and PDW showed a decrease. Histologically, we confirmed that platelet counts reached stable levels after completion of lung alveolarization in rats (at around postnatal day 20). Correlation analysis demonstrated that platelet counts in rats were significantly associated with the MLI, a marker of alveolar size.

As far as we know, this is the first time to demonstrate an association between platelet index and perinatal lung development. We found a persistent increase in PLT and an overall decrease of MPV and PDW during the first 8 weeks of life of extremely premature infants, which was partly supported by the study from Henry et al., who showed a stepwise increase in platelets during the first 3 months of life in newborn infants (14). Notably, the authors also showed that newborns with advancing gestational age had higher platelet levels at birth (14), suggesting that the platelet parameters are dynamically changing with development during early life. This study also showed a correlation between platelet indexes and lung alveolarization in rats. The lung is an important organ in platelet biogenesis. In humans and rodents around 50% of circulating platelets are generated in the lung (8, 10–12). In the lung, platelets arise in the vascular bed by shedding from megakaryocytes and pro-platelets which embolize in the lungs. Shear stress, turbulence, and endothelial interaction in the pulmonary vascular bed play an important role in platelet biogenesis by activating shedding from megakaryocytes and pro-platelets (9). During lung development the size of the vascular bed increases, thereby increasing the capacity of platelet synthesis and secretion into the systemic circulation. This explains at least in part the gradual increase in PLT during normal postnatal lung development. Two elegant studies have demonstrated that platelets contribute to embryonic lung development (6) and lung regeneration after pneumonectomy (7), which may explain why the decrease of platelets led to blunted lung development in the current study.

This study showed that platelet counts are significantly lower in infants developing BPD and in rats with experimental BPD induced by hyperoxia. This finding is in line with a study by Okur et al. who reported a lower PLT and PMI in BPD infants during the first week of life (15), but is not supported by Go et al. who report that platelet parameters at birth were not associated with BPD after multivariate analysis (16). This difference might be due to the different timing of these studies. Common pulmonary diseases in adults, including asthma, chronic obstructive pulmonary disease, idiopathic pulmonary fibrosis, and pulmonary hypertension, are not associated with reduced platelet counts (9). This is probably caused by redundancy of the adult pulmonary vascular bed. Only in severe cases of acute respiratory distress syndrome thrombocytopenia was observed, caused by either increased platelet consumption or decreased platelet production (17). PLT largely depend on platelet synthesis and consumption, in which the lung plays an important role. We speculate that in BPD systemic PLT are low because (I) the production of platelets is decreased due to a reduced vascular bed caused by aberrant alveolar and vascular development and lung injury, and (II) increased platelet consumption in the injured lung exposed to hyperoxia.

**Figure 6:**
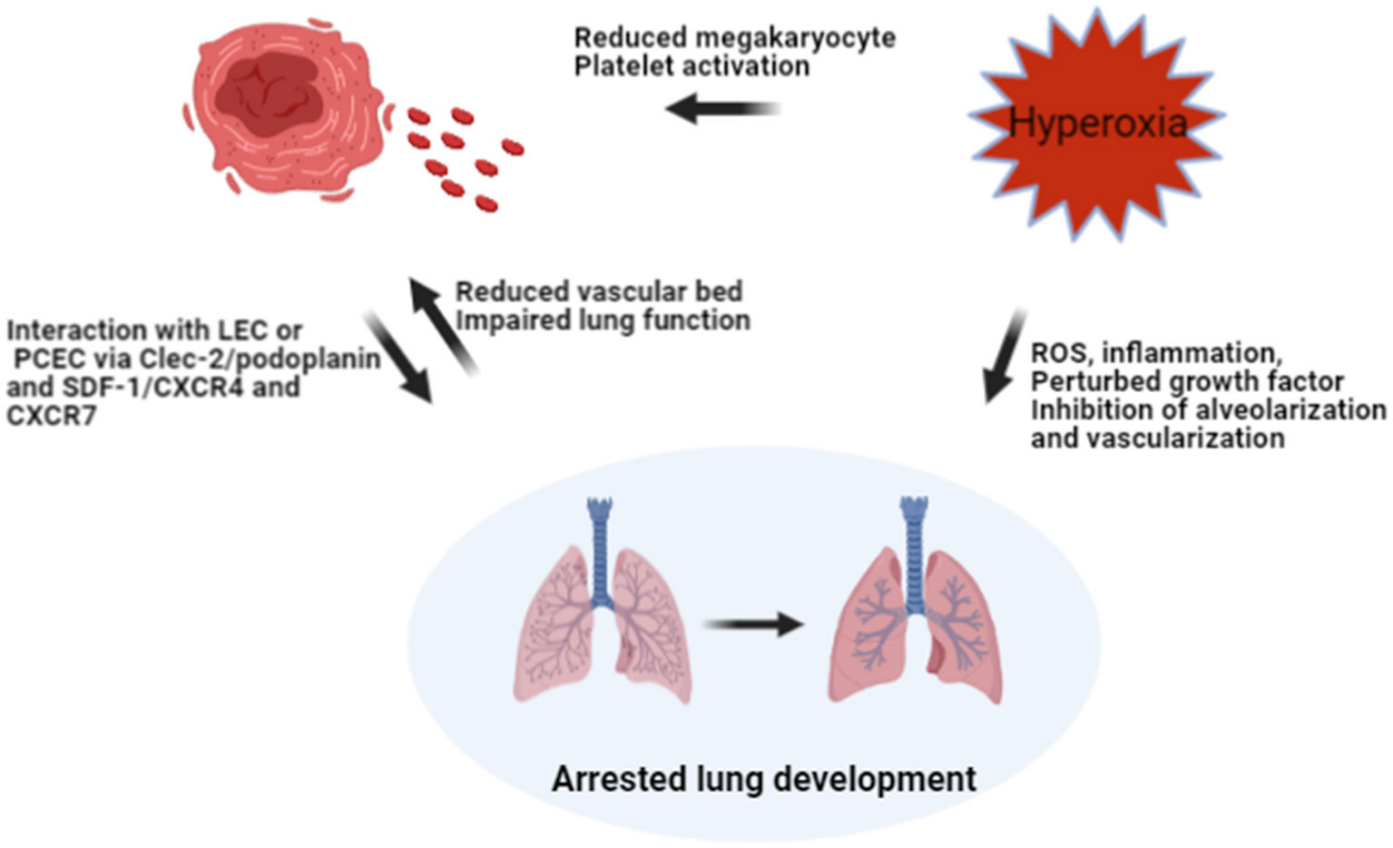
Hypothesis on the interaction between hyperoxia, platelet biogenesis, and lung injury. Hyperoxia may reduce the number of megakaryocytes in the lung and increase platelet consumption by activating platelets, leading to decreased platelet count directly. Hyperoxia may also impair the developing lung by inhibiting alveolarization and vascularization, leading to BPD. The reduction of the vascular bed in BPD, together with impaired lung function might result in reduced shedding of megakaryocytes and pro-platelets trapped in pulmonary capillaries. Meanwhile, reduced PLT may hamper lung development by interacting with LEC and PCEC via ligands and receptors. ROS, reactive oxygen species; LEC, lymphatic endothelial cells; PCEC, pulmonary capillary endothelial cells; Clec-2, C-type lectin-like receptor-2; SDF-1, stromal-cell-derived factor-1; CXCR4 and CXCR7, receptors of SDF-1 on PCEC.

Previous studies have shown that platelet production may be regulated by oxygen tension. Acute hypoxia in rodents led to a biphasic response with an initial increase, followed by a decrease in platelet counts after 1 week of hypoxia, which may be caused by hypoxia-induced hemoconcentration, platelet activation and vasoconstriction (9, 18, 19). Hyperoxia decreases platelet counts by inhibiting platelet production and enhancing platelet activation for thrombi formation and platelet consumption (20–22). Therefore, there may be a vicious cycle linking platelet production and blunted lung development in the BPD setting (Figure 6). However, platelet production and postnatal lung development are both evolving processes, so it is hard to speculate whether decreased platelet biogenesis contributes to BPD, or BPD leads to the reduction of platelets. Well-designed experimental studies are needed to elucidate this interaction.

The study by Henry et al. and our study found a transient increase of MPV in infants during their first 2 weeks of life and then a decrease, which is independent of gestational age at birth (14). However, these findings were inconsistent with the studies from Dani et al. and Cekmez et al. who reported that infants developing BPD had higher MPV levels at 1–3 days of life compared to those without BPD (23, 24). This discrepancy might be attributed to the different time points that platelet parameters were measured. MPV and PDW are both indicators of platelet size, indicating platelets are being produced or activated. In the animal experiment, we observed an increase in MPV and PDW in oxygen-exposed rats, which might indicate more platelets are activated in these pups. In BPD infants, we also observed a lower platelet count and a higher MPV at postnatal week 2, which might indicate more platelets are activated in these infants. Besides, we also observed a physiological increase in MPV in these infants, the mechanism under this phenomenon still needs investigation. We speculate that platelet parameters in the first days after birth are prone to be affected by the neonatal transition and medical treatments. Therefore, the analysis of platelet parameters over the clinically defined time window of BPD may provide more reliable evidence.

There are several limitations to this study. The most relevant one is that the direct correlation between impaired platelet formation and arrested lung development was not studied. Besides, we could not evaluate the effect of hyperoxia itself on platelet parameters in extremely premature infants due to the complexity of their clinical condition. Moreover, platelet parameters in rats exposed to hyperoxia were only measured on day 10. It would be interesting to track the interaction between platelet parameters and lung development for a longer period.

In conclusion, clinical and experimental BPD are significantly associated with lower platelet levels. The physiological increase of platelet counts synchronizes with postnatal alveolarization in neonatal rats. Understanding the role of platelets in neonatal lung development may shed new light on BPD prevention in clinics.

## Data Availability Statement

The raw data supporting the conclusions of this article will be made available by the authors, without undue reservation.

## Ethics Statement

The studies involving human participants were reviewed and approved by the Institutional Ethical Committee of Shenzhen Maternity and Child Healthcare Hospital. Written informed consent for participation was not provided by the participants' legal guardians/next of kin because: Acquirement of informed consent was waived given that no personal data were explicitly reported. The animal study was reviewed and approved by the Institutional Animal Care and Use Committee of Shenzhen Institutes of Advanced Technology of the Chinese Academy of Sciences.

## Author Contributions

CY, FW, and XC conceptualized and designed the study and wrote the first draft of the manuscripts. XC, JZho, and FY carried out the clinical data collection. XC and JZha performed the data analysis. GW, CY, and FW reviewed and revised the manuscripts. All authors read and approved the final manuscript.

## Conflict of Interest

The authors declare that the research was conducted in the absence of any commercial or financial relationships that could be construed as a potential conflict of interest.
